# Embracing cohort heterogeneity in clinical machine learning development: a step toward generalizable models

**DOI:** 10.1038/s41598-023-35557-y

**Published:** 2023-05-24

**Authors:** Michiel Schinkel, Frank C. Bennis, Anneroos W. Boerman, W. Joost Wiersinga, Prabath W. B. Nanayakkara

**Affiliations:** 1 Center for Experimental and Molecular Medicine (CEMM), Location Academic Medical Center, Amsterdam UMC Location University of Amsterdam, Meibergdreef 9, 1105 AZ Amsterdam, The Netherlands; 2 Quantitative Data Analytics Group, Department of Computer Science, Vrije Universiteit Amsterdam, De Boelelaan 1105, Amsterdam, The Netherlands; 3 Department of Internal Medicine, Section General Internal Medicine, Amsterdam UMC Location Vrije Universiteit Amsterdam, De Boelelaan 1117, Amsterdam, The Netherlands; 4 Department of Internal Medicine, Amsterdam UMC University of Amsterdam, Meibergdreef 9, Amsterdam, The Netherlands

**Keywords:** Computational biology and bioinformatics, Computational models, Data processing, Predictive medicine

## Abstract

This study is a simple illustration of the benefit of averaging over cohorts, rather than developing a prediction model from a single cohort. We show that models trained on data from multiple cohorts can perform significantly better in new settings than models based on the same amount of training data but from just a single cohort. Although this concept seems simple and obvious, no current prediction model development guidelines recommend such an approach.

Hospitals nowadays collect vast amounts of data, far exceeding what physicians can process^1^. There is a growing interest in artificial intelligence (AI) models to analyze these data in real-time and provide decision support. However, current AI models often show poor generalizability to new settings. A prime example is a widely implemented sepsis detection model, which has substantial performance drops in practice and burdens hospitals across the United States with alert fatigue^2^.

Differences in AI model performance between hospitals often result from variations in case mix (population heterogeneity) and local protocols or used devices (operational heterogeneity)^3^. Traditionally, medical AI models are trained on a single cohort, which increases the chances that the model will fit those hospital-specific patterns. We hypothesize that AI models trained on multiple cohorts, adding heterogeneity and diluting hospital-specific patterns, are more generalizable to other settings, which has also been suggested previously in various studies^4,5^. The current study aims to compare the performance of single versus multicohort trained prediction models and uses our recently developed blood culture prediction tool as an example^6^. In that study, we extracted general laboratory results and vital sign measurements of patients who had a blood culture drawn during their emergency department stay in one of included centers. We then used these data to train a machine learning model to predict the target of whether the blood culture would become positive or negative (the latter included likely contaminants). The Amsterdam University Medical Centers’ (UMC) local medical ethics review committee waived the review of the current study as the Act of Research with Human Subjects did not apply (IRB number: IRB00002991; case: 2020.486). All methods were carried out in accordance with local guidelines and (privacy) regulations, and the need for informed consent was waived due to the deidentified nature of the data.

The data for this study were derived from our previous study of patients undergoing blood culture draws in the emergency department of the VU University Medical Center (VUMC), Zaans Medical Center (ZMC), and Beth Israel Deaconess Medical Center (BIDMC). Details on the cohorts can be found elsewhere^6^. We trained a traditional, single-cohort-based model to predict blood culture outcomes (6000 VUMC patients) and validated it in the two others. We also trained models on mixed, more heterogeneous data while keeping the training size equal (e.g., 3000 VUMC/3000 ZMC patients or 3000 VUMC/3000 BIDMC patients) and validated them in the remaining cohort (Fig. 1). The model development is described in the Supplementary Appendix. We compare the areas under the curve (AUCs) of the various sets of predictions and estimate the 95% confidence interval around the differences in AUC using bootstrap resampling with replacement in 10.000 samples.

**Figure 1:**
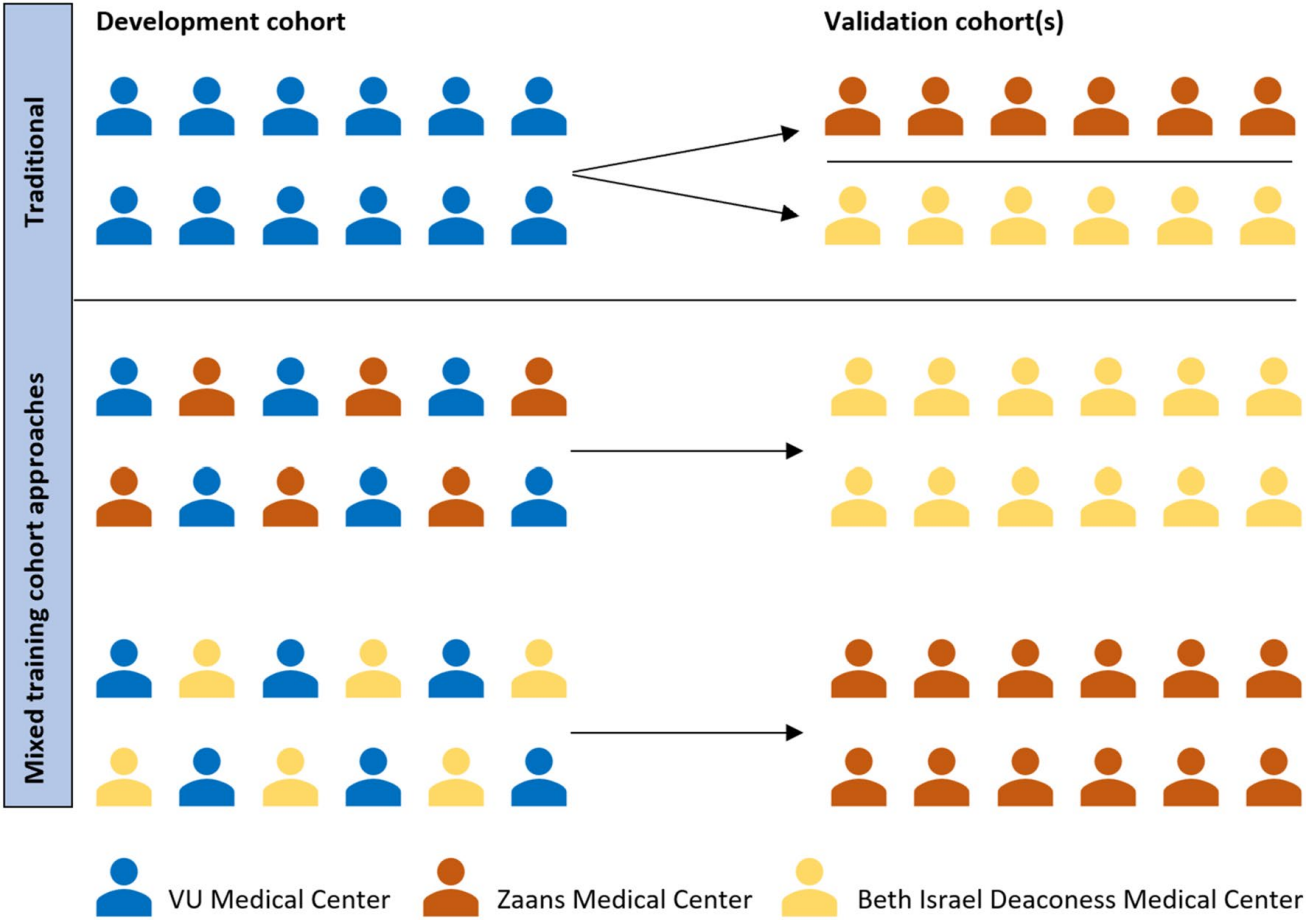
A visual representation of the compositions of traditional and mixed training cohort approaches as used in our study. The traditional approach uses only one cohort to train a prediction model and validates in one or more external cohorts. The mixed training cohort approach uses multiple datasets to train a prediction model, increasing heterogeneity and diluting hospital-specific patterns. Consequently, the model may better capture genuinely disease-specific predictors, which can significantly improve the performance in external validation cohorts.

When trained on data from two cohorts (VUMC and ZMC), our model reaches an AUC of 0.756 in the complete BIDMC cohort (n = 27.706; Fig. 2a), significantly outperforming the traditional single-cohort approach trained on VUMC data (AUC = 0.739; Fig. 2b). The difference between the model is 0.017 (95% CI 0.011 to 0.024). The calibration plot of the traditional approach does show a better calibration curve, with a slope round 1, while the multicohort model seems to be overconfident in rare cases with higher probabilities.

**Figure 2:**
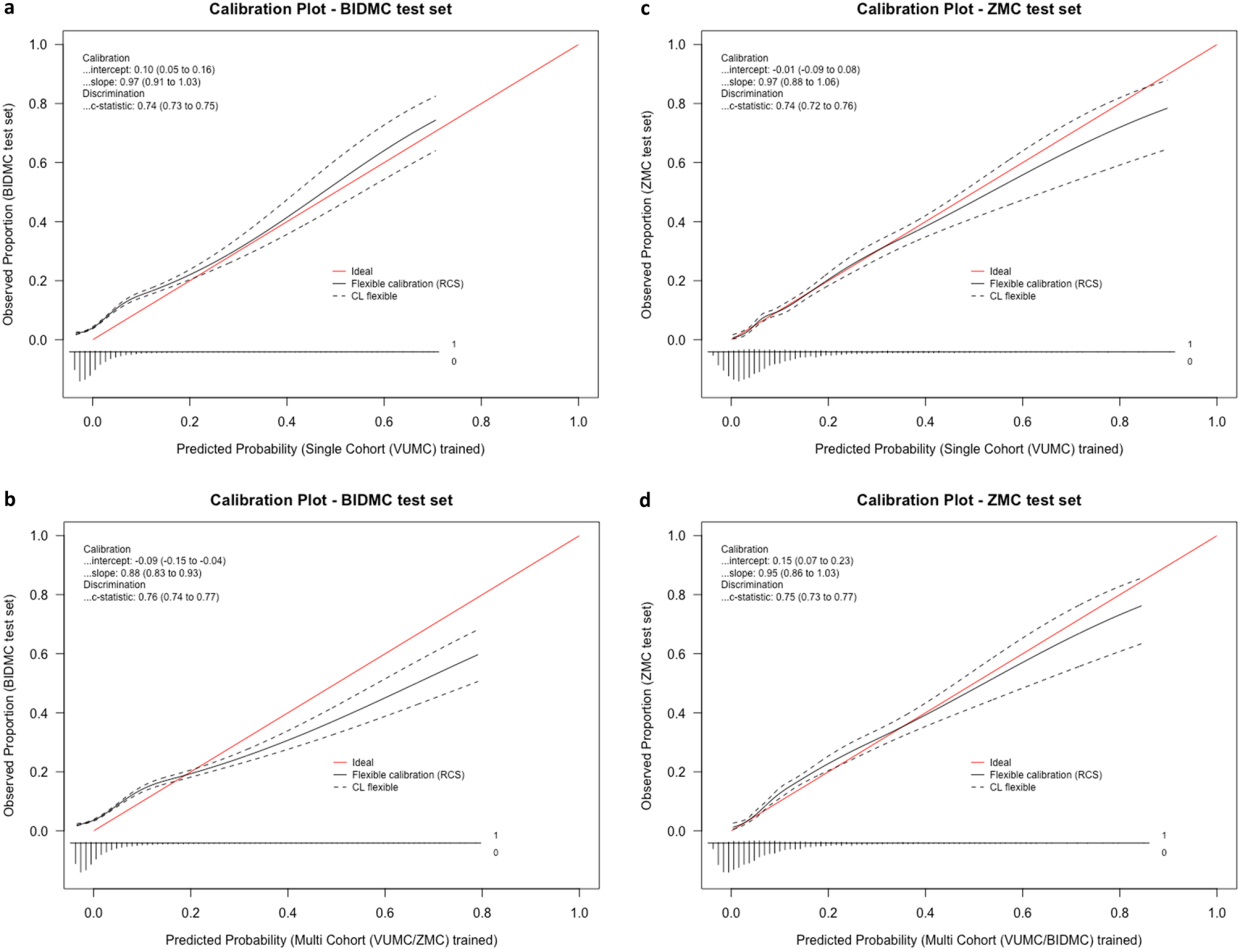
Comparing calibration plots and Areas Under the Curve (AUCs; c-statistics) of prediction models trained on mixed populations compared with a traditional approach trained on a single population. We use our recently developed prediction tool for the outcomes (positive or negative) of a collected blood culture in the emergency department^6^. (**a**) Shows the calibration in the complete BIDMC cohort (n = 27.706) of a traditional blood culture prediction model trained on 6000 VUMC patients. (**b**) Shows the same calibration plot in the BIDMC cohort of a model trained on a mixed cohort of 3000 VUMC and 3000 ZMC patients. (**c**) Shows the calibration in the complete ZMC cohort (n = 5.961) of a traditional model trained on 6000 VUMC patients. (**d**) Shows the same calibration plot in the BIDMC cohort of a model trained on a mixed cohort of 3000 VUMC and 3000 BIDMC patients. *VUMC* VU University Medical Center, *ZMC* Zaans Medical Center, *BIDMC* Beth Israel Deaconess Medical Center.

A model using VUMC and BIDMC data reaches an AUC of 0.752 when tested in the ZMC cohort (n = 5.961; Fig. 2c). While higher than the AUC of the traditional single-cohort model trained on VUMC data (AUC = 0.742; Fig. 2d), the difference is non-significant (0.010; 95% CI − 0.002 to 0.023). Both models seem to be well-calibrated in this dataset.

Combining cohorts to diversify training data can significantly improve the generalizability of medical prediction models. By diluting cohort-specific patterns, models may better detect disease-specific predictors. This could provide significant benefits in large-scale clinical implementations as it may limit performance drops, such as observed with the sepsis detection algorithm^2^. Although it has been suggested that this problem could also be restricted by validating and recalibrating models for use in new settings, our approach will be more valuable for implementation in smaller hospitals, which may not have the resources to recalibrate a model^7^.

Notably, performances of the traditional and mixed models in the ZMC cohort did not differ significantly, perhaps due to the smaller sample size. Alternatively, combining two exceptionally different cohorts, such as VUMC (Netherlands) and BIDMC (United States), may make finding disease-specific predictors more challenging, despite a dilution of cohort-specific patterns. The tradeoff between training cohort similarity and heterogeneity should be carefully considered. On top of that, it is even more important to consider calibration beyond the AUC curves when using models trained on mixed cohorts. We observed a worse calibration in one of the multicohort models, which could be for example be caused by differing baseline risks for a positive blood culture, which need to be addressed during modeling procedures.

In conclusion, these data on a specific prediction task show that a model trained on combined cohorts reach significantly higher AUC scores in a new setting, which makes intuitive sense, but is not yet recommended by established development guidelines. The increasing numbers of publicly available datasets, such as the BIDMC data, make it feasible to use multiple cohorts for medical AI development^8^. We encourage researchers to explore the simple yet effective approach of combining cohorts to improve generalizability to new settings, while being cautious of model calibration issues.

## Supplementary Information

Supplementary Information.

## Data Availability

The datasets used and/or analyzed during the current study are available from the corresponding author on reasonable request.
