# Supplementary material for: Embracing cohort heterogeneity in clinical machine learning development: a step toward generalizable models
Source: Sci Rep. 2023 May 24;13:8363. doi: 10.1038/s41598-023-35557-y (PMC10209202; doi:10.1038/s41598-023-35557-y)
Supplement: Supplementary file 1 — Supplementary Information. [file 41598_2023_35557_MOESM1_ESM.docx]

# Supplementary appendix

### Methods - Study design

The retrospective study which captured the data used in this analysis adhered to the “transparent reporting of a multivariable prediction model for individual prognosis or diagnosis (TRIPOD).” The Amsterdam University Medical Centers’ local medical ethics review committee waived the study review (IRB number: IRB00002991; case: 2020.486), as the medical research involving Human Subjects Act did not apply.

### Methods - study populations

The de-identified data used in this analysis are identical to the data captured in the original study. It includes patients who were 18 years or older and underwent a blood culture draw in the emergency department of the Dutch VU University Medical Center (VUMC; between 2016-2021), the Dutch Zaans Medical Center (ZMC; between 2016-2021), or the American Beth Israel Deaconess Medical Center (BIDMC; between 2011-2019). Data of the BIDMC was made available through the MIMIC-IV-ED database, freely available to researchers worldwide (https://physionet.org/content/mimic-iv-ed/1.0).

### Methods – model development

From the complete cohorts, training cohorts of 6000 or 3000 patients were randomly selected, stratified by blood culture outcomes. The validations were always based on the complete cohort of a particular center. We trained several eXtreme Gradient Boosting (XGBoost) classifiers based on the various combinations of cohorts. The optimal hyperparameters were found through small-scale fivefold cross-validated grid searches for learning rate (0.1, 0.3, 0.5), minimal child weight (1, 5, 10), and maximum tree depth (1, 3, 5).

### Methods – data analysis

Model development was performed using Python version 3.8.1, including the sklearn, xgboost, numpy, pandas, and matplotlib libraries. The calibration plots were made in R version 4.2.3 using the CalibrationCurves package and the val.prob.ci.2() function. Bootstrapping was performed with the ROCR and boot packages and resampling with replacement in 10.000 samples.
